# Estimation of mechanical properties of Mg-5Zn-0.5Al-xSn alloy based on virtual crystal approximation

**DOI:** 10.1016/j.heliyon.2022.e11224

**Published:** 2022-10-26

**Authors:** Yu Zhang, Bo Wang, Shicheng Wei, Yujiang Wang, Linwei Li

**Affiliations:** National Key Laboratory for Remanufacturing, Army Academy of Armored Forces, Beijing 100072, PR China

**Keywords:** First-principles calculations, Virtual crystal approximation, Multi-component Mg alloys, Mechanical properties

## Abstract

First-principles calculations of multi-component alloys have been studied in detail. Herein, the first-principles calculations of Mg-5Zn-0.5Al-xSn alloys were performed by using the virtual crystal approximation (VCA) method. By calculating the lattice constants and elastic constants of the Mg-5Zn-0.5Al-xSn doping models, it was found that the mechanical properties and micro-hardness were related with the content of Sn. With the increase of Sn content, and the best ductility and the smallest micro-hardness were achieved at Sn = 2 wt.%. To verify the calculation results, the Mg-5Zn-0.5Al-xSn alloys were prepared and micro-hardness and tensile tests were conducted. The experiments demonstrate that the trends in mechanical properties obtained from the experiments are in agreement with the VCA computational results. These findings indicate that the VCA method has guiding significance in industries for rapid screening of high-performance Mg alloys.

## 1 Introduction

As “the green engineering materials in the 21st century” [1], Mg alloys have many advantages, such as rich reserves, light weight and high strength [2], [3], but the comprehensive mechanical properties of pure Mg are poor [4], [5]. At present, alloying is usually used to improve the mechanical properties of Mg alloys, and common alloy elements include Zn, Al, Sn, Ca, Mn, etc [4], [6]. Among them, Zn and Al have good solid solution strengthening effect on Mg alloys [7], and Sn can effectively improve ductility when used with Al and improve the compressive strength of the alloys [8], therefore Mg-Zn-Al-Sn alloy has become a hot research topic [9], [10].

At present, the research on Mg-Zn-Al-Sn alloy mostly adopts the traditional trial and error research based on experience [9], [10], [11], [12], Zhou et al. [11] studied the tensile properties of Mg-6Zn-4Al-xSn alloys through experiments, and Ding et al. [12] studied the corrosion behavior of Mg-5Zn-4Al-xSn alloys through experiments. However, the traditional experimental method is time-consuming and laborious to study the Mg-Zn-Al-Sn alloys. Therefore, the first-principles calculation is considered to guide the experimental design, so as to quickly analyze the influence of the content of Sn on the mechanical properties of the alloy.

However, a multi-component system has a large volume, and its supercell model is difficult to converge when first-principles calculations are employed. The virtual crystal approximation (VCA) is a computational method for calculating mixed systems in first-principles calculations, which is an effective method to study the effect of micro change of components on properties [13], [14]. For the properties of Mg alloys, Cui et al. [15] used the VCA method to study binary Mg alloys Mg_1-x_Zn_x_, and the calculated results are in good agreement with those of other experimental works. Al Hagan et al. [16] calculated Zn_1-x_Mg_x_Se using VCA, and the calculated elastic constants are consistent with the experiments. However, VCA method have not been used in Mg-Zn-Al-Sn alloys yet.

Herein, the VCA method based on first-principles calculations was used for Mg-5Zn-0.5Al-xSn. The effect of Sn addition on the mechanical properties of the alloy was studied by constructing models of Mg alloys with different contents of Sn. Micro-hardness and tensile tests were conducted on the prepared samples to verify the calculation results and evaluate the reliability and guiding significance of first-principles calculations in the estimation of mechanical properties of multi-component Mg alloy.

## 2 Materials and methods

**Figure 1:**
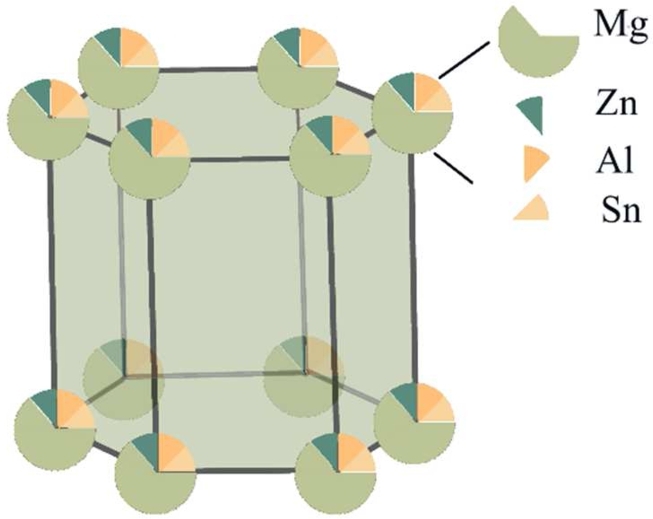
VCA model crystal structure diagram.

The first-principles calculations were based on the density functional theory (DFT) with the plane-wave cutoff energy of 540 eV and 24×24×24 Monkhorst–Pack k-point grid. Ultra-soft pseudo potentials (USPPs) were used to represent the interactions between ionic cores and valence electrons. The Broyden-Fletcher-Goldfarb-Shanno (BFGS) method was employed to optimize the crystal structure until the total energy changes converged to $5.0\times 10^{-7}$ eV/atom. For the exchange-correlation energy calculations, Generalized gradient approximation (GGA) was used with Perdew, Burke, and Ernzerh of approach. The VCA method was used to approximate the doping of Mg-5Zn-0.5Al-xSn alloys. The total pseudopotential was calculated as the different elements of Mg alloys in proportion. The VCA model was constructed as a Hexagonal Close Packed (HCP) structure, as shown in the Fig. 1.

As Mg has a hexagonal close-packed structure (HCP), the lattice constant is a=b; and when c/a=1.633, the crystal has the closest stacking structure [17], [18]. Therefore, in this paper, *a*, *c* and c/a were mainly studied.

According to Hooke's law, the elastic stiffness constant, $C_{ij}$ (GPa) and elastic compliance constant $S_{ij}$ (GPa^−1^), as Eq. (1), (2).

(1) $$\sigma_{i}=C_{ij}\varepsilon_{j},\;i,j=1,2,3,4,5,6$$

(2) $$\varepsilon_{i}=S_{ij}\sigma_{j},\;i,j=1,2,3,4,5,6$$

where $\sigma_{i}$ is the stress and $\varepsilon_{i}$ is the strain.

The hexagonal crystal system has five independent elastic stiffness tensors, namely, $C_{11}$, $C_{33}$, $C_{44}$, $C_{12}$ and $C_{13}$. According to the symmetry proposed by Voigt [19], other elastic stiffnesses are calculated as Eq. (3).

(3) $$C_{21}=C_{12},C_{31}=C_{13}=C_{32},C_{22}=C_{11},C_{55}=C_{44},C_{66}=\frac{1}{2}(C_{11}-C_{12})$$

For the hexagonal crystal system, the system stability needs to meet the following conditions [20]:

(4) $$C_{44}>0,C_{11}>|C_{12}|,C^{2}=(C_{11}+2C_{12})C_{33}-2C_{13}^{2}>0$$

The bulk modulus, $K_{V}$, and shear modulus, $G_{V}$, were calculated using Voigt model [19], as follows:

(5) $$K_{V}=\frac{1}{9}[\left(C_{11}+C_{22}+C_{33}\right)+2\left(C_{12}+C_{23}+C_{31}\right)]=\frac{1}{9}(2C_{11}+C_{33}+2C_{12}+4C_{13})$$

(6) $$G_{V}=\frac{1}{15}\left[\left(C_{11}+C_{22}+C_{33}\right)-\left(C_{12}+C_{23}+C_{31}\right)+3\left(C_{44}+C_{55}+C_{66}\right)\right]=\frac{7}{30}C_{11}+\frac{1}{15}C_{33}-\frac{1}{6}C_{12}-\frac{2}{15}C_{13}+\frac{2}{5}C_{44}$$

The bulk modulus, $K_{R}$, and shear modulus, $G_{R}$, were calculated using the elastic compliance constant, $S_{ij}$, as per the Reuss model [21], as follows:

(7) $$K_{R}={\left[\left(S_{11}+S_{22}+S_{33}\right)+2\left(S_{12}+S_{23}+S_{31}\right)\right]}^{-1}=\frac{C^{2}}{C_{11}+C_{22}+2C_{33}-4C_{13}}$$

(8) $$G_{R}=15{\left[4\left(S_{11}+S_{22}+S_{33}\right)-4\left(S_{12}+S_{23}+S_{31}\right)+3\left(S_{44}+S_{55}+S_{66}\right)\right]}^{-1}=\frac{5}{2}\left[\frac{C_{22}C_{44}C_{66}}{3B_{V}C_{44}C_{66}+C^{2}\left(C_{44}+C_{66}\right)}\right]$$

The bulk modulus, $K_{H}$, shear modulus, $G_{H}$, Young's modulus, *E*, and Poisson's ratio, *υ* were calculated using the Hill model [22], as follows:

(9) $$K_{H}=\frac{1}{2}\left(K_{V}+K_{R}\right)$$

(10) $$G_{H}=\frac{1}{2}\left(G_{V}+G_{R}\right)$$

(11) $$E=\frac{9K_{H}G_{H}}{3K_{H}+G_{H}}$$

(12) $$\upsilon =\frac{3K_{H}-2G_{H}}{2\left(3K_{H}+G_{H}\right)}$$

The Mg-5Zn-0.5Al-xSn (x = 0, 0.5, 1, 2, 3) alloy ingots were prepared from pure Mg (99.95%), Zn (99.99%), Al (99.95%), Sn (99.95%) in a resistance furnace 740∼760 °C. The mixed atmosphere of CO_2_ and SF_6_ (CO_2_: SF_6_ = 6:1) was used as protective gas. The preheated Mg ingots were added in batches. The power was turned off to start the alloying procedure and pure Zn, Al, and Sn were added while the melt was stirred to achieve a uniform composition. After refining, the power was cut off to allow the system to cool naturally to 740 °C. The slag casting was performed and several Φ 80 mm ingots were obtained. These ingots were extruded through an XJ-800t horizontal extruder at the extrusion temperature of 300 °C, extrusion ratio of 25:1, and extrusion speed of 1 m/min to obtain Φ 16 mm bars.

The composition of alloys was characterized using the inductively coupled plasma optical emission spectrometer (ICP-OES, Optima 8300) and Scanning Electron Microscope (SEM, Quanta 250 FEG). The micro-hardness was tested with a microhardness tester (TH 765) with uploading of 50 g and dwell time of 15 s. Each sample was repeated fifteen times and average was calculated. The tensile tests of alloys were performed using an electronic universal testing machine (CMT-5105) at the strain rate of $10^{-3}\cdot$s^−1^ at room temperature. In the tensile tests, each sample was repeated three times to ensure accuracy. The samples used for tensile tests had the gage length of 25 mm and gage cross-sectional diameter of Φ 5 mm.

## 3 Results and discussion

### 3.1 First-principles calculation results

The modeling and structure optimization of Mg were conducted, and the calculated lattice constants and elastic constants of Mg are listed in Table 1.

**Table 1:** Lattice parameters and elastic constants of Mg.

Reference	*a*/Å	*c*/Å	*c*/*a*	*C*_11_/GPa	*C*_33_/GPa	*C*_44_/GPa	*C*_12_/GPa	*C*_13_/GPa	Method
This work	3.198	5.19	1.623	66.995	66.442	18.073	22.074	21.977	GGA+PBE
Ref. [23]	3.209	5.211	1.624	-	-	-	-	-	Experiment at 25 °C
Ref. [24]	3.203	5.200	1.624	-	-	-	-	-	Experiment at 25 °C
Ref. [15]	3.210	5.240	1.632	-	-	-	-	-	Abinit
Ref. [25]	-	-	-	63.48	66.45	18.42	25.94	21.70	Experiment at 0K
Ref. [26]	-	-	-	58	66	20	30	22	GGA+PBE

Comparing the crystal model of Mg developed in this work (see Table 1) with the experimental values as Ref. [23], [24], the errors in *a*, *c*, and c/a were all within 0.19%, and compared with the calculation results as Ref. [15], the errors in *a*, *c*, and c/a were all within 0.95%, indicating that the calculation result is relatively accurate. Comparing with the experimental values of Ref. [25], the errors of this work in $C_{11}$, $C_{33}$, $C_{44}$, $C_{12}$ and $C_{13}$ were 5.54%, 0.01%, 1.88%, 14.90%, and 1.28%, respectively, while Ref. [26] reported respective values as 8.63%, 0.68%, 8.58%, 15.65%, 1.38%, respectively, indicating the calculated results of this work are more consistent with the experimental results. Therefore, the optimized Mg model demonstrates that the parameters, methods, and results used in the calculations were reliable, and the model can be used to develop the VCA model.

The VCA method was used to construct the doping models of Mg-5Zn-0.5Al-xSn (x = 0, 0.25, 0.5, 1, 2, and 3). The structure was optimized, the elastic constants were calculated, and the mechanical properties of each model were obtained as per Eq. (1)–(12). The results are listed in Table 2.

**Table 2:** Calculated results of ZAT50.5x.

	ZAT50.50	ZAT50.50.25	ZAT50.50.5	ZAT50.51	ZAT50.52	ZAT50.53
*a*/Å	3.184	3.183	3.184	3.182	3.186	3.186
*c*/Å	5.207	5.207	5.210	5.203	5.191	5.173
*c*/*a*	1.635	1.636	1.636	1.635	1.630	1.624
*C*_11_/GPa	72.167	71.925	68.924	64.938	61.090	66.814
*C*_33_/GPa	71.641	72.150	72.219	70.933	71.862	75.859
*C*_44_/GPa	17.633	17.450	15.956	14.571	18.239	19.180
*C*_12_/GPa	22.404	23.497	26.745	31.587	37.155	33.768
*C*_13_/GPa	20.761	20.278	19.703	21.127	21.581	22.683
*K*_*V*_/GPa	38.314	38.234	38.041	38.721	39.408	40.862
*G*_*V*_/GPa	22.233	21.953	20.195	17.628	17.271	19.667
*K*_*R*_/GPa	38.312	38.221	38.022	38.692	39.376	40.856
*G*_*R*_/GPa	21.545	21.272	19.425	16.880	15.855	19.050
*K*_*H*_/GPa	38.313	38.228	38.031	38.706	39.392	40.859
*G*_*H*_/GPa	21.889	21.612	19.810	17.254	16.563	19.359
*E*/GPa	55.162	54.556	50.638	45.066	43.581	50.155
*υ*	0.260	0.262	0.278	0.306	0.316	0.295
*K*_*H*_/*G*_*H*_	1.750	1.769	1.920	2.243	2.378	2.111
$G_{H}^{3}/K_{H}^{2}$	7.145	6.908	5.375	3.428	2.928	4.345

All the elastic constants of doping models satisfy Eq. (4), $C_{44}>0,C_{11}>|C_{12}|,C^{2}=(C_{11}+2C_{12})C_{33}-2C_{13}^{2}>0$, (see Table 2), implying that the doping models had good mechanical stability. Since the c/a of the ideal HCP crystal is ${\left(8/3\right)}^{1/2}\approx 1.633$, the closer the c/a is to 1.633, the tighter the structure [17], [18]. With the rise in Sn, the c/a tended to decrease and the difference with 1.633 tended to increase, indicating that the tightness of the doping models decreased gradually. With the increase in Sn, *E* first decreased and reached the minimum at Sn = 2 wt.%. As $K_{H}/G_{H}$ is a common parameter reflecting the brittleness and ductility of materials, when $K_{H}/G_{H}>1.75$, the materials displayed ductility, otherwise displayed brittleness [27], [28]. In this work, *υ* and $K_{H}/G_{H}$ increased with the rise in Sn, and $K_{H}/G_{H}>1.75$, signifying that the Mg alloys displayed ductility. With the increase in Sn, ductility enhanced and reached the maximum at Sn = 2 wt.%. Therefore, with the increase in Sn, the comprehensive mechanical properties of Mg-5Zn-0.5Al-xSn gradually improved and became optimal at Sn = 2 wt.%. As reported by Chen [3], micro-hardness is $H_{V}\propto G^{3}/K^{2}$; with the increase in Sn content, $H_{V}$ decreased first and then increased. The micro-hardness was the lowest at Sn = 2 wt.%. As per VCA, the comprehensive mechanical properties of Mg-5Zn-0.5Al-xSn gradually enhanced with the rise in Sn content and reached the best at Sn = 2 wt.%. Therefore, the calculation shows that the content of Sn is related to the mechanical properties of Mg-Zn-Al-Sn alloy, and the research of Ref. [29], [30] also proves this.

### 3.2 Experimental results

In order to further explain the influence of Sn content on the micro-hardness and mechanical properties of Mg alloys, Mg-5Zn-0.5Al-xSn are prepared to verify. Table 3 displays that the composition of the designed alloys was consistent with the actual composition of the alloys after smelting, indicating that the amount of burning loss was small and the prepared alloys can validate the impact of Sn on the mechanical properties of Mg-5Zn-0.5Al-xSn (x = 0, 0.5, 1, 2, and 3) alloys.

**Table 3:** Chemical composition of Mg-5Zn-0.5Al-xSn alloys (wt.%).

Alloy	Zn (wt.%)	Al (wt.%)	Sn (wt.%)	Mg (wt.%)
Mg-5Zn-0.5Al-0Sn(ZAT50.50)	5.41	0.53	0.00	Bal.
Mg-5Zn-0.5Al-0.5Sn (ZAT50.50.5)	5.00	0.51	0.51	Bal.
Mg-5Zn-0.5Al-1Sn (ZAT50.51)	4.99	0.50	1.01	Bal.
Mg-5Zn-0.5Al-2Sn (ZAT50.52)	4.98	0.50	2.05	Bal.
Mg-5Zn-0.5Al-3Sn (ZAT50.53)	4.99	0.50	3.03	Bal.

**Figure 2:**
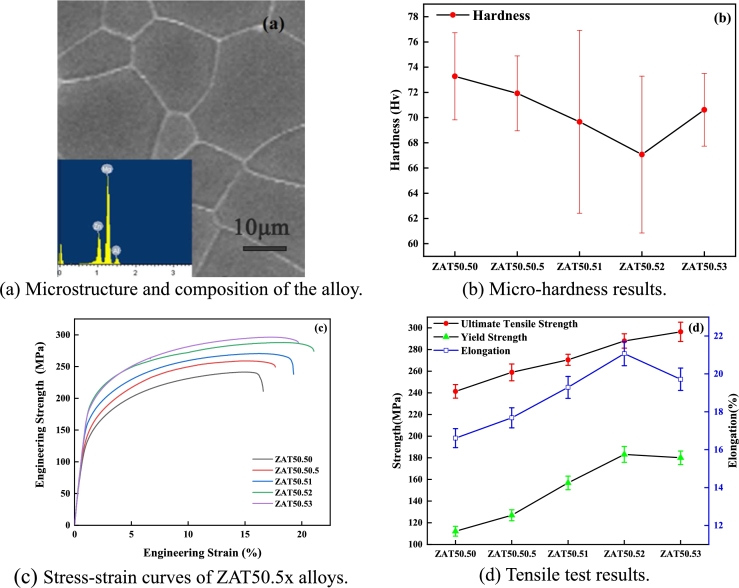
Experimental results of ZAT50.5x alloys.

Fig. 2(a) presents the SEM-EDS result of Mg-5Zn-0.5Al-0Sn. The figure shows the solid solution state of the material as a whole, and the composition contained all the four elements, namely Mg, Zn, Al, and Sn. Therefore, the mechanical properties were tested excluding the effect of precipitated phases. Fig. 2(b) displays the micro-hardness test results, and the results illustrate that the overall micro-hardness decreased and then increased, with the lowest micro-hardness of 67.07 HV at Sn = 2 wt.%. The stress-strain tensile curve is shown in Fig. 2(c). To quantify the experimental results, the specific experimental data is presented in Fig. 2(d).

As per Fig. 2(d), with the increase in Sn, the ultimate tensile strength (UTS), yield strength (YS), and elongation (EL) demonstrated an upward trend. As per the above results, the comprehensive properties were the best with UTS = 287.91 MPa, YS = 183.13 MPa, and EL = 21.07% at Sn = 2 wt.%. Comparing the calculation results of VCA and the experimental results of micro-hardness, UTS, YS, and EL were consistent with the simulation results, signifying that the VCA method can guide the design of multi-component Mg alloys.

## 4 Conclusion

VCA calculation was applied to calculate the trend in mechanical properties of Mg-5Zn-0.5Al-xSn alloys. Mg-5Zn-0.5Al-xSn alloys were prepared to validate the simulation results. The calculated results of VCA were consistent with the experimental results. Micro-hardness of Mg-5Zn-0.5Al-xSn alloys decreased first and then increased as the addition of Sn decreased, and reached the minimum as the addition of Sn was 2 wt.%. The mechanical properties gradually increased as the addition of Sn decreased. Mg-5Zn-0.5Al-xSn alloy showed the best mechanical properties when the addition of Sn was 2 wt.%, and UTS, YS, EL were, 287.91 MPa, 183.13 MPa, 21.07%, respectively. Therefore, the VCA method can be effectively employed for the design of Mg alloys, which can aid to achieve a significant reduction in the calculation and experimental period.

## Declarations

### Author contribution statement

Yu Zhang: Conceived and designed the experiments; Performed the experiments; Analyzed and interpreted the data; Wrote the paper.

Bo Wang: Performed the experiments; Analyzed and interpreted the data; Wrote the paper.

Shicheng Wei: Conceived and designed the experiments; Contributed reagents, materials, analysis tools or data.

Yujiang Wang: Contributed reagents, materials, analysis tools or data.

Linwei Li: Performed the experiments; Analyzed and interpreted the data.

### Funding statement

This work was supported by 10.13039/501100001809National Natural Science Foundation of China [51905543], National Defense Science and Technology Excellence Young Scientists Foundation [2017-JCJQ-ZQ-001].

### Data availability statement

Data included in article/supplementary material/referenced in article.

### Declaration of interests statement

The authors declare no conflict of interest.

### Additional information

No additional information is available for this paper.
